# Molecular analysis of 3D domain swapping in the acylphosphatase from *Escherichia coli*

**DOI:** 10.1107/S2059798326001774

**Published:** 2026-03-19

**Authors:** Sergio Martínez-Rodríguez, Jose A. Gavira, M. Carmen Salinas-Garcia, Montserrat Andujar-Sánchez, Ana Camara-Artigas

**Affiliations:** a Department of Biochemistry and Molecular Biology III and Immunology University of Granada Avenida Doctor Jesús Candel Fábregas 11 18071Granada Spain; b Raw Materials, Human and Environmental Health, Associated Unit of the CSIC by the IACT–CSIC, CSIC–Universidad de Granada, Avenida de las Palmeras 4, 18100Armilla, Spain; c Laboratorio de Estudios Cristalográficos, CSIC, Avenida de las Palmeras 4, 18100Armilla, Spain; d Department of Chemistry and Physics University of Almeria, Agrifood Campus of International Excellence (ceiA3) and CIAMBITAL Carretera de Sacramento s/n 04120Almeria Spain; e Department of Physical Chemistry University of Granada Avenida Fuentenueva 18071Granada Spain; Lund University, Sweden

**Keywords:** acylphosphatases, proline isomerization, 3D domain swapping, amyloid aggregation

## Abstract

Three-dimensional domain swapping is a mechanism by which proteins form oligomers. At present, the molecular basis that dictates whether some proteins fold in their monomeric form or as intertwined oligomers is poorly understood. Previously, we have described the first intertwined dimer of an acylphosphatase (AcP) from crystals belonging to the orthorhombic space group *C*222. In this work, we present the first crystallographic structure of monomeric AcP from *Escherichia coli* (EcoAcP) and compare it with the intertwined structure of the orthorhombic polymorph and a new intertwined dimer structure obtained from crystals belonging to the hexagonal space group *P*6_1_22. The monomeric form contains two molecules in the asymmetric unit, each exhibiting some differences. One of the molecules shows a sodium cation that introduces conformational changes in loop L4 (connecting α2 and β4). This loop is located adjacent to the active site, which is formed by a cleft between loops L1 (connecting β1 and α1) and L3 (connecting β2 and β3), in which phosphate anions have been modelled. Besides, in the monomeric form, the active-site Arg20 forms a salt bridge with the carboxyl-terminal group. This interaction is absent in the intertwined dimer, where the interchange of the terminal β-strand β5 is facilitated by loop L5 (connecting β4 and β5) that serves as a hinge loop. The first residue of this loop, Pro79, has been modelled in a *cis* conformation in the intertwined structures, whereas it is in a *trans* conformation in the monomeric form. The low rate of *cis*–*trans* proline isomerization would favour the formation of the domain-swapped structure under appropriate conditions. Comparative analysis of the monomer and intertwined dimer structures would facilitate understanding of the molecular basis of oligomer formation.

## Abstract 2

Structures of the monomer and intertwined dimer of the acylphosphatase from *E. coli* shed light on the molecular basis of its 3D domain swapping.

## 1. Introduction

Acylphosphatase (AcP; EC 3.6.1.7) is a small enzyme of ∼100 residues that catalyses the hydrolysis of acyl–phosphate bonds in acyl phosphates. The enzyme is widespread, being found from archaebacteria to humans. The 3D structure features a ferredoxin-like fold, comprising a five-stranded antiparallel β-sheet that faces two α-helices. The small size of the enzyme and its propensity to form amyloid fibrils at acidic pH values have made AcP an ideal model to study protein folding and misfolding (Motamedi-Shad *et al.*, 2009 ▸; Taddei *et al.*, 1999 ▸; Chiti *et al.*, 1998 ▸, 2000 ▸, 2001 ▸; van Nuland *et al.*, 1998 ▸; Calamai *et al.*, 2009 ▸; Bemporad *et al.*, 2004 ▸, 2008 ▸; Chiti, Webster *et al.*, 1999 ▸; Parrini *et al.*, 2008 ▸). Understanding the mechanism of amyloid aggregation is crucial for comprehending the molecular basis of various neurodegenerative diseases. Many proteins implicated in amyloid formation have also been reported to undergo three-dimensional domain swapping (3D-DS; Cámara-Artigas, 2016 ▸; Janowski *et al.*, 2001 ▸; Staniforth *et al.*, 2001 ▸; Kumar *et al.*, 2023 ▸). However, to date, the only AcP that has been proven to undergo 3D-DS is the *Escherichia coli* AcP (EcoAcP; Martínez-Rodríguez *et al.*, 2023 ▸).

EcoAcP (UniProt code P0AB65) presents an unusual thermal stability, behaving more like a hyperthermophilic AcP than a mesophilic AcP (Ramazzotti *et al.*, 2006 ▸). EcoAcP and the hyperthermophilic AcPs share a longer fifth loop (L5; residues 80–88 connecting β4 and β5), which is not conserved among AcPs. Besides, an atypical intrachain disulfide bridge between Cys5 and Cys49 has been suggested to be responsible for the higher rate of folding and stability of EcoAcP compared with other AcPs (Parrini *et al.*, 2008 ▸). This disulfide bridge is also present in the structures of *Vibrio cholerae* AcP (VchAcP; UniProt code A5F8G9; PDB entry 6krb; Chatterjee *et al.*, 2020 ▸) and *Coxiella burnetii* AcP (CbuAcP; UniProt code Q83AB0; PDB entry 3trg; Franklin *et al.*, 2015 ▸). In EcoAcP, the disulfide bond links the N-terminal region of the protein and the fourth loop (L4; residues 64–71 connecting α2 and β4).

Studies on the enzymatic activity of AcPs concluded that arginine and asparagine residues in the active site are responsible for substrate binding and catalysis, respectively (Stefani *et al.*, 1997 ▸). The phosphate-binding site of EcoAcP reveals a similarity between its phosphate-binding loop (Val14-Gln-Gly-Val-*X*-*X*-Arg20) and that previously reported in the structures of different enzymes involved in the biochemical utilization of phosphate-containing substrates, including the critical residue Asn38 involved in catalysis.

In this work, we present the first crystal structure of the monomeric form of EcoAcP and a new crystal form of the intertwined dimer. We have compared these structures with the known monomeric structure, as determined by NMR (PDB entry 2gv1; Pagano *et al.*, 2006 ▸), and the previously reported intertwined dimer (PDB entry 8bv9; Martínez-Rodríguez *et al.*, 2023 ▸). Comparison of the monomeric and dimeric forms of EcoAcP enabled us to identify the conformational changes in the hinge loop and other regions of the protein that may favour the formation of intertwined dimers. Additionally, the high-resolution structure of the monomeric form enabled us to identify several new features that may regulate the activity of this small enzyme.

## 2. Materials and methods

All chemicals were of analytical grade and were used without further purification. Ni–NTA agarose was purchased from Qiagen. *E. coli* BL21(DE3) was used for overexpression. EcoAcP was overexpressed and purified as described previously (Martínez-Rodríguez *et al.*, 2023 ▸).

### 2.1. Protein crystallization

Crystallization screenings were performed using the vapour-diffusion technique with a sitting-drop setup at 20°C, employing Crystal Screen and Crystal Screen 2 from Hampton Research. 1 µl freshly purified recombinant C-terminally His_6_-tagged EcoAcP protein solution (10–12 mg ml^−1^ in 20 m*M* Tris–HCl pH 8.0, 50 m*M* NaCl) was mixed with an equal volume of precipitant solution from the reservoir. Two conditions were identified to yield EcoAcP crystals and optimized to improve the size and quality of the crystals: (i) 0.2 *M* ammonium sulfate, 30%(*w*/*v*) PEG 4000 pH 4.8 and (ii) 20% PEG 10 000, 0.1 *M* HEPES pH 7.5.

### 2.2. Data collection and refinement

Prior to data collection, crystals were harvested from the crystallization drop using LithoLoops (Molecular Dimensions, Sheffield, UK), transferred to a 5 µl drop of mother solution containing 15%(*v*/*v*) glycerol as a cryoprotectant (<60 s) and flash-cooled in liquid nitrogen. Diffraction data were collected at 100 K on the BL13-XALOC beamline at the ALBA synchrotron, Barcelona, Spain (Juanhuix *et al.*, 2014 ▸) and the ID30A-3 beamline at the ESRF synchrotron, Grenoble, France (McCarthy *et al.*, 2018 ▸). Data were indexed and processed using the *XDS* software (Kabsch, 1976 ▸) and scaled with *AIMLESS* from the *CCP*4 suite (Agirre *et al.*, 2023 ▸) within the *autoPROC* toolbox (Vonrhein *et al.*, 2011 ▸). The data-collection statistics are shown in Table 1 ▸.

The *Phenix* suite was used to solve the structures (Liebschner *et al.*, 2019 ▸). Molecular-replacement phasing was performed using the *Phaser-MR* GUI in *Phenix* (Afonine *et al.*, 2012 ▸). The coordinates of the intertwined dimeric (PDB entry 8bv9) and monomeric (PDB entry 2gv1) forms of EcoAcP were used as models for molecular replacement. The final models of the monomeric and dimeric forms were obtained after several manual building cycles in *Coot* (Emsley & Cowtan, 2004 ▸; Emsley *et al.*, 2010 ▸). Water molecules were automatically modelled using *phenix.refine* in *Phenix* (Afonine *et al.*, 2012 ▸) and manually inspected in the difference electron-density maps. In the final rounds of refinement, some molecules from the precipitant solution were modelled. The final models were validated using *MolProbity* and *PDB-REDO* (Chen *et al.*, 2010 ▸; Joosten *et al.*, 2012 ▸). The structure-solution and refinement statistics are shown in Table 2 ▸.

### 2.3. Sequence and structure analysis

**Figure 1:**
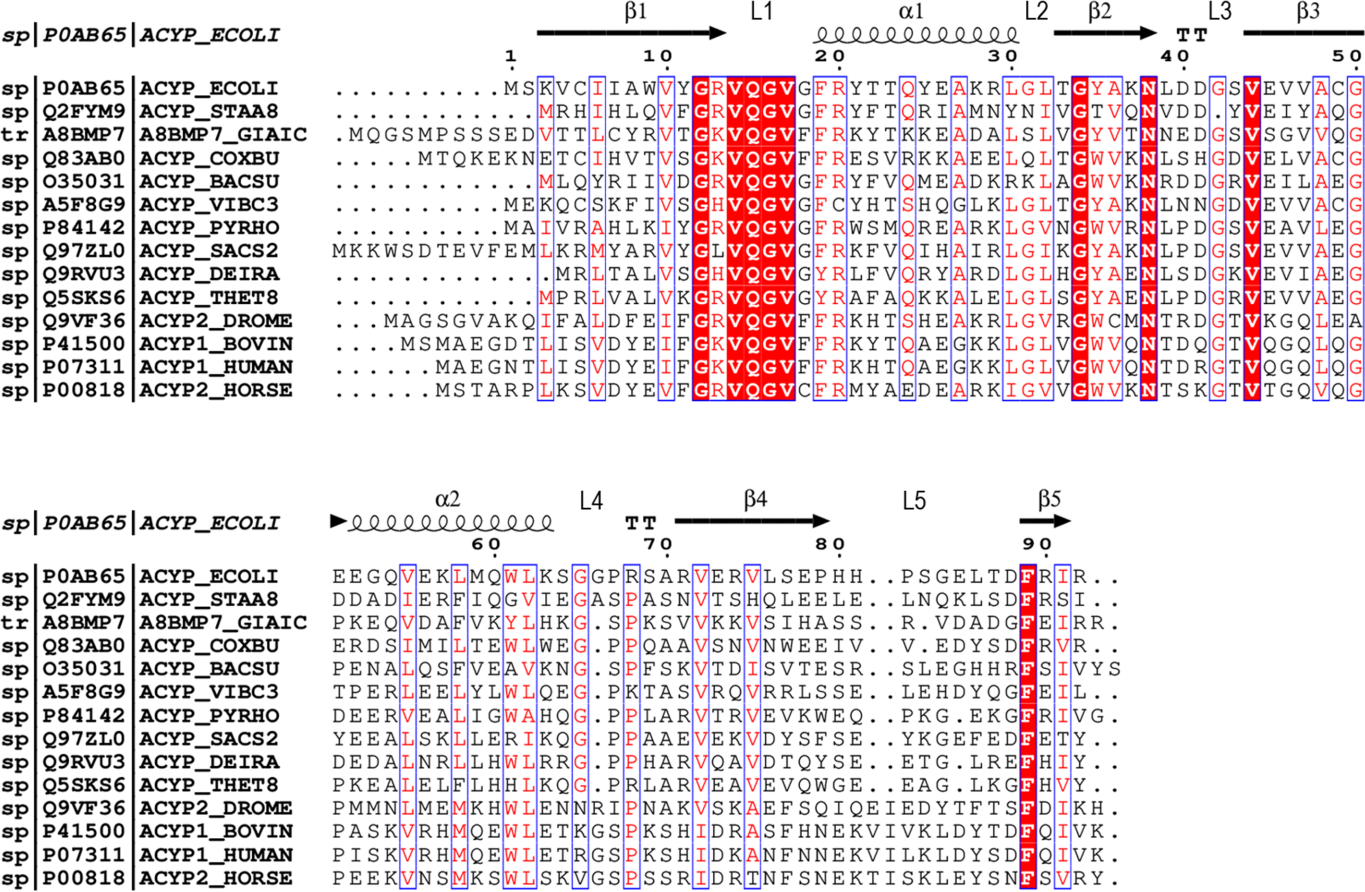
Sequence alignment of AcPs with 3D structures available in the PDB. Each sequence is indicated by its UniProt code. The secondary structure of EcoAcP is indicated in the upper part of the sequence. Conserved residues among AcP sequences are coloured red.

*Clustal Omega* (Madeira *et al.*, 2019 ▸) and *ESPript* (Robert & Gouet, 2014 ▸) were used for multiple sequence alignment. Fig. 1 ▸ shows the alignment of EcoAcP (P0AB65) with the sequences of AcPs that have an experimental 3D structure available. The secondary-structure elements of the crystallo­graphic structure of EcoAcP were determined using *PROMOTIF* (Hutchinson & Thornton, 1996 ▸) in the *PDBsum* web server (Laskowski *et al.*, 2018 ▸). Structure superposition and r.m.s.d. calculations were performed using the *LSQKABCCP*4 module (Kabsch, 1976 ▸). The protein interfaces in the crystal were analysed using the *PISA* server (Krissinel, 2011 ▸). Distances between amino acids were calculated using the *CONTACT* program of the *CCP*4 suite (Krissinel *et al.*, 2022 ▸). The presence of metals in the crystallographic structure was checked using the *CheckMyMetal* web server (Gucwa *et al.*, 2023 ▸). Hydrogen-bond and accessible surface-area (ASA) analyses were performed with the *VADAR* server (Willard *et al.*, 2003 ▸). Figures were generated using the *PyMOL* 3.1.1 software (version 1.8; Schrödinger).

## 3. Results

### 3.1. Protein crystallization and structure of the monomeric form of EcoAcP

**Figure 2:**
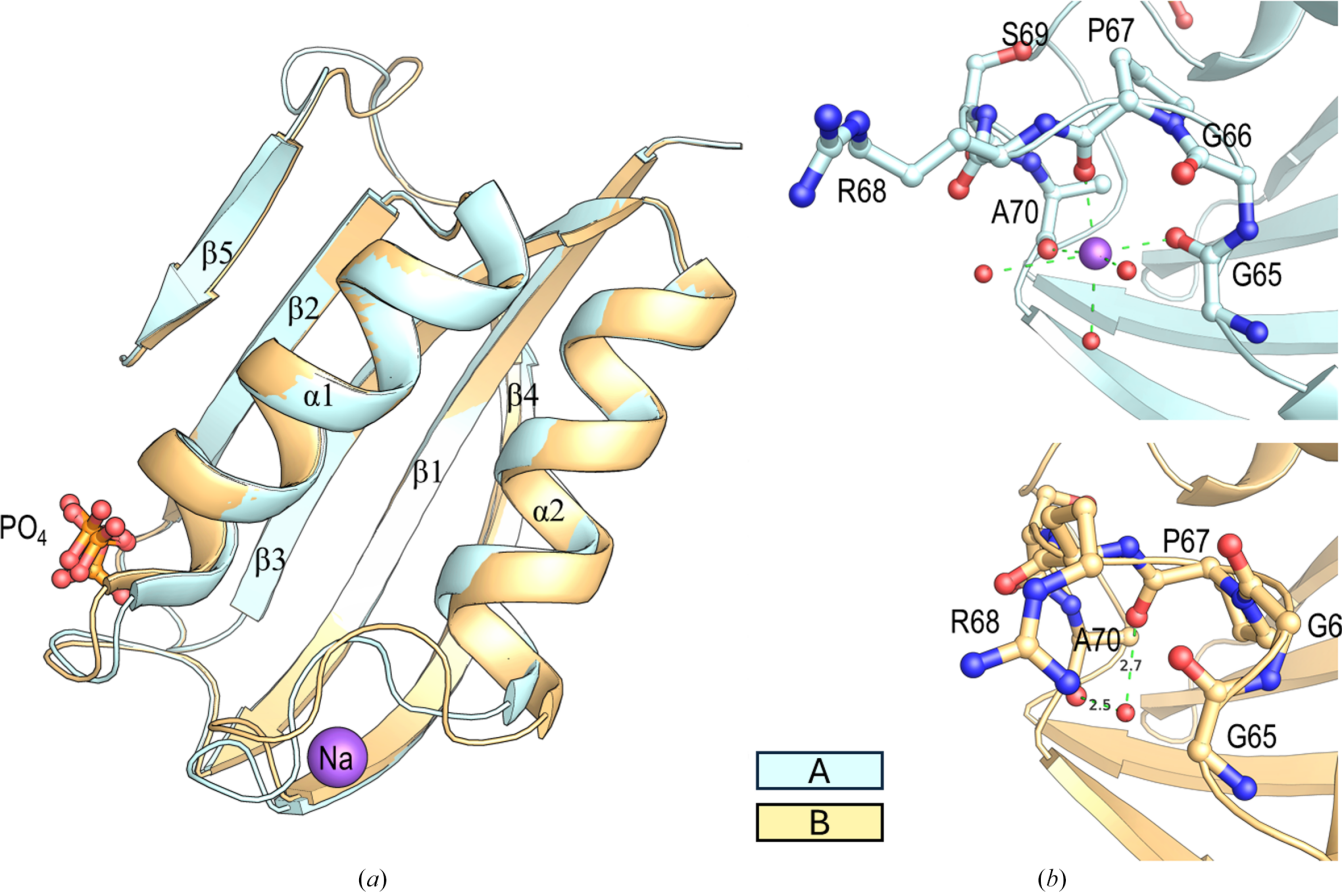
(*a*) Cartoon representation of the superposition of chains *A* and *B* of monomeric EcoAcP. (*b*) Loop L4 in chains *A* and *B* is shown in stick representation. In chain *A*, interactions of residues around the sodium ion (purple sphere) are represented as green dashed lines. In chain *B*, a water molecule occupies the position of the sodium ion.

The crystal structure of the monomeric form of EcoAcP was obtained using 20% PEG 10 000, 0.1 *M* HEPES pH 7.5 as the precipitant. The crystals belonged to the ortho­rhombic space group *P*2_1_2_1_2_1_, with unit-cell parameters *a* = 28.30, *b* = 48.58, *c* = 103.04 Å. Two molecules of monomeric EcoAcP were modelled in the asymmetric unit. The overall fold of EcoAcP is composed of five β-strands (β1, 2–13; β2, 33–39; β3, 43–51; β4, 71–80; β5, 89–92) and two helices (α1, 18–31; α2, 52–64). As previously reported, Cys5 and Cys49 form a disulfide bridge. Although the superposition of the two chains in the asymmetric unit results in a backbone r.m.s.d. value of 0.21 Å, there are noticeable differences between the chains (Fig. 2 ▸*a*). In chain *A*, a sodium ion has been modelled bound to the carbonyl O atom of residues Gly65, Pro67, Ala70 and three water molecules. The distances between the carbonyl O atoms of the three amino acids and the sodium ion are ∼2.3 Å, which is characteristic, together with the octahedral geometry, of the presence of an Na atom bound to the protein. The presence of this sodium ion was validated using the *CheckMyMetal* web server (Gucwa *et al.*, 2023 ▸), which is consistent with the atomic distances and geometry of the surrounding atoms. This Na atom is not present in chain *B*, where the loop connecting α2 with β4 (loop L4) shows a different conformation (Fig. 2 ▸*b*).

**Figure 3:**
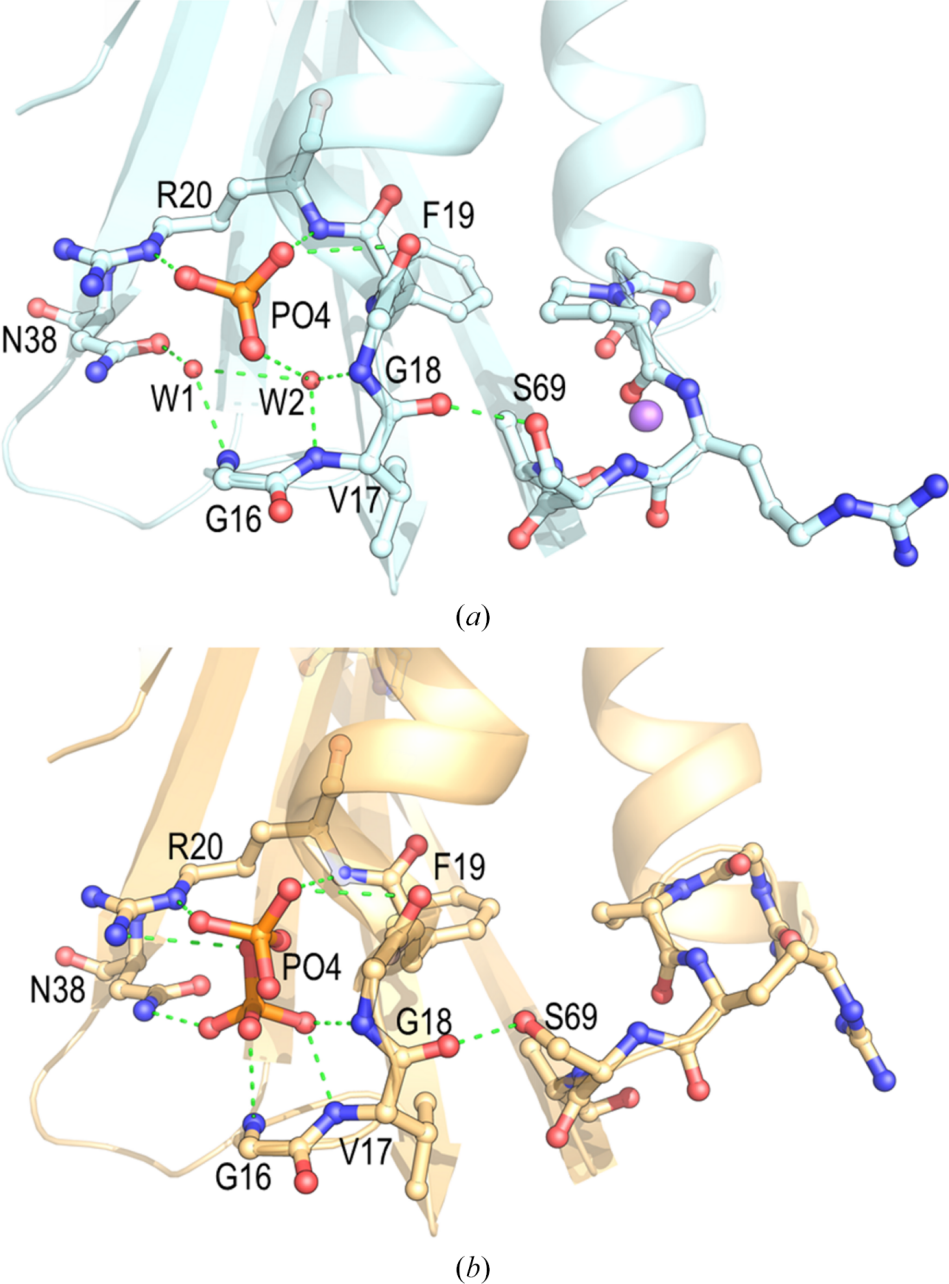
Catalytic site of monomeric EcoAcP. (*a*) In chain *A*, residues interacting with the phosphate ion are represented as sticks, and the bonding interactions are shown as green dashed lines. Two water molecules are placed in the catalytic site at hydrogen-bond distances to residues implicated in catalysis. (*b*) Chain *B* shows two alternate conformations of the phosphate ion.

Different acyl phosphates are substrates of this enzyme, and several crystallographic structures show a phosphate ion modelled in the active site: *Pyrococcus horikoshii* AcP (PhoAcP; PDB entries 3tnv and 2w4d; Lam *et al.*, 2011 ▸), *Sulfolobus solfataricus* AcP (SsoAcP; PDB entry 4ojg; de Rosa *et al.*, 2014 ▸), *Deinococcus radiodurans* AcP (DraAcP; PDB entry 8jfs; Khakerwala *et al.*, 2023 ▸), *Bacillus subtilis* AcP (BsuAcP; PDB entries 2hlt and 3br8) and a mutant of human common-type AcP (HsaAcP; PDB entry 3toq). In other structures, a sulfate ion has been modelled at the same position: *Sulfolobus solfataricus* AcP (SsoAcP; PDB entry 2bjd; Corazza *et al.*, 2006 ▸) and *Vibrio chlolerae* AcP (PDB entry 6krb; Chatterjee *et al.*, 2020 ▸). The active site of EcoAcP is located at the cleft between loop L1 (connecting β1 and α1) and L3 (connecting β2 and β3). Residues Arg13–Arg20 from L1 create a cradle-like conformation, in which the backbone N atoms of Gly16, Val17, Gly18, Phe19 and Arg20 are pointing toward its centre. The sequence of loop L1 is mainly conserved (Fig. 1 ▸) and shows nearly the same conformation in both chains. A single phosphate ion has been modelled in chain *A*, bound to Arg20 and Gly18, along with a water molecule (W1), at a suitable distance from Asn38 (Fig. 3 ▸). This configuration likely represents the nucleophile assisting substrate hydrolysis, as previously suggested (see, for example, Cheung *et al.*, 2005 ▸; Stefani *et al.*, 1997 ▸). On the other hand, the electron density in the difference maps in chain *B* allowed the phosphate ion to be modelled in two alternate conformations. One of the conformations is equivalent to the phosphate found in chain *A* (Fig. 3 ▸), while the other phosphate ion is placed in the proposed environment that could be occupied by the acyl group of the substrate (Cheung *et al.*, 2005 ▸). Interestingly, the diverse positions of the phosphate ion modelled in the active sites of other AcP structures resemble the two alternate conformations found in EcoAcP chain *B*. Considering the catalytic mechanism proposed for bovine testis AcP (Thunnissen *et al.*, 1997 ▸), the two alternate conformations of the phosphate group found in EcoAcP most likely show (i) a plausible binding position of the phosphatidyl and acetyl moieties of the acetyl phosphate substrate and/or (ii) a mimic of the transition state of catalysis (Supplementary Fig. S1).

### 3.2. The role of Arg20 in the thermophilic characteristics of EcoAcP

**Figure 4:**
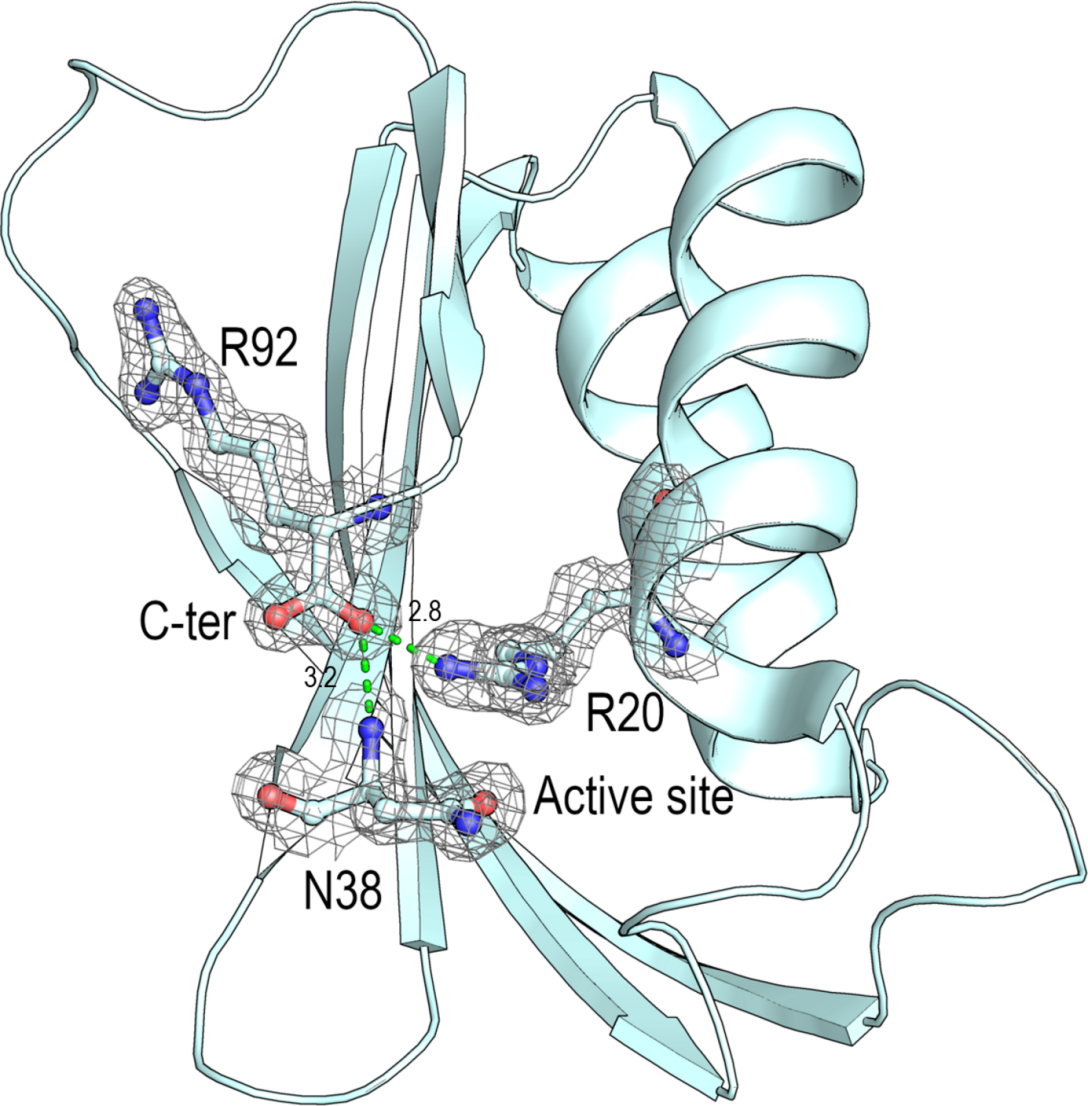
2*F*_o_ − *F*_c_ electron-density map contoured to 1σ, showing the interactions between the carboxyl-terminal group of Arg92 and the catalytic residues Arg20 and Asn38 (green dashed lines) in the monomeric structure of EcoAcP (PDB entry 9sv1).

Unexpectedly, whereas the recombinant EcoAcP was produced containing the terminal residues LEHHHHHH, the high resolution of the monomeric EcoAcP structure clearly reveals proteolysis of the tag and the formation of a salt bridge between the active-site Arg20 and the carboxyl-terminal group of Arg92 (Fig. 4 ▸). This salt bridge has also been found in the hyperthermophilic PhoAcP, which shows reduced catalytic efficiency at 25°C. The presence of this salt bridge has been proposed to increase the rigidity of Arg20 in the active site by locking the guanidinium group, thereby preventing conformational fluctuation during catalysis. This salt bridge is absent in mesophilic AcPs and is primarily responsible for the observed differences in the temperature dependence of enzymatic activity between thermophilic and mesophilic AcPs. The presence of this salt bridge has been proposed to confer the observed thermophilic character on EcoAcP (Lam *et al.*, 2011 ▸).

In addition to effects on enzymatic activity and stability, electrostatic interactions between the carboxyl group of Arg92 and the side chains of Arg20 and Asn38 in this structure would protect the polypeptide from the opening observed in the intertwined dimer, resulting in the monomeric form of EcoAcP. This finding is relevant, as the last residue in the EcoAcP sequence is Arg92 (UniProt code P0AB65).

### 3.3. Structure of the intertwined dimer of EcoAcP in a hexagonal space group

Crystals of the EcoAcP intertwined dimer were obtained at 20°C using 0.2 *M* ammonium sulfate, 30%(*w*/*v*) polyethylene glycol 4000 pH 4.8 as the precipitant solution. This crystal belonged to space group *P*6_1_22, with unit-cell parameters *a* = 90.8, *b* = 90.8, *c* = 40.3 Å, and shows some differences from the orthorhombic polymorph previously described at 2.55 Å resolution (PDB entry 8bv9; Martínez-Rodríguez *et al.*, 2023 ▸). As in the previous intertwined structure, the hexagonal polymorph of EcoAcP has a single chain of the protein present in the asymmetric unit, and a symmetry operator generates the dimer. This hexagonal polymorph diffracted to higher resolution (1.95 Å), enabling more accurate modelling of the protein and solvent. Although several sulfate ions from the precipitant solution have been modelled, none of them are placed in the active site. The residues forming the active site exhibit higher *B* factors than in the monomeric crystal structure and the previous dimeric form, which showed a bound sulfate ion. It has been reported that the flexibility of the active site is critical for substrate binding and enzyme activity. Indeed, the NMR structure of BsuAcP (PDB entry 2hlu) reveals some disorder at the active-site loop L1 (residues Gly14–Arg18), whereas it converted to an ordered state upon binding phosphate anion (PDB entry 2hlt) (Hu *et al.*, 2010 ▸; Supplementary Fig. S2). Similar findings have been made for the AcP from *S. solfataricus* (SsoAcP; Pagano *et al.*, 2006 ▸, 2010 ▸).

**Figure 5:**
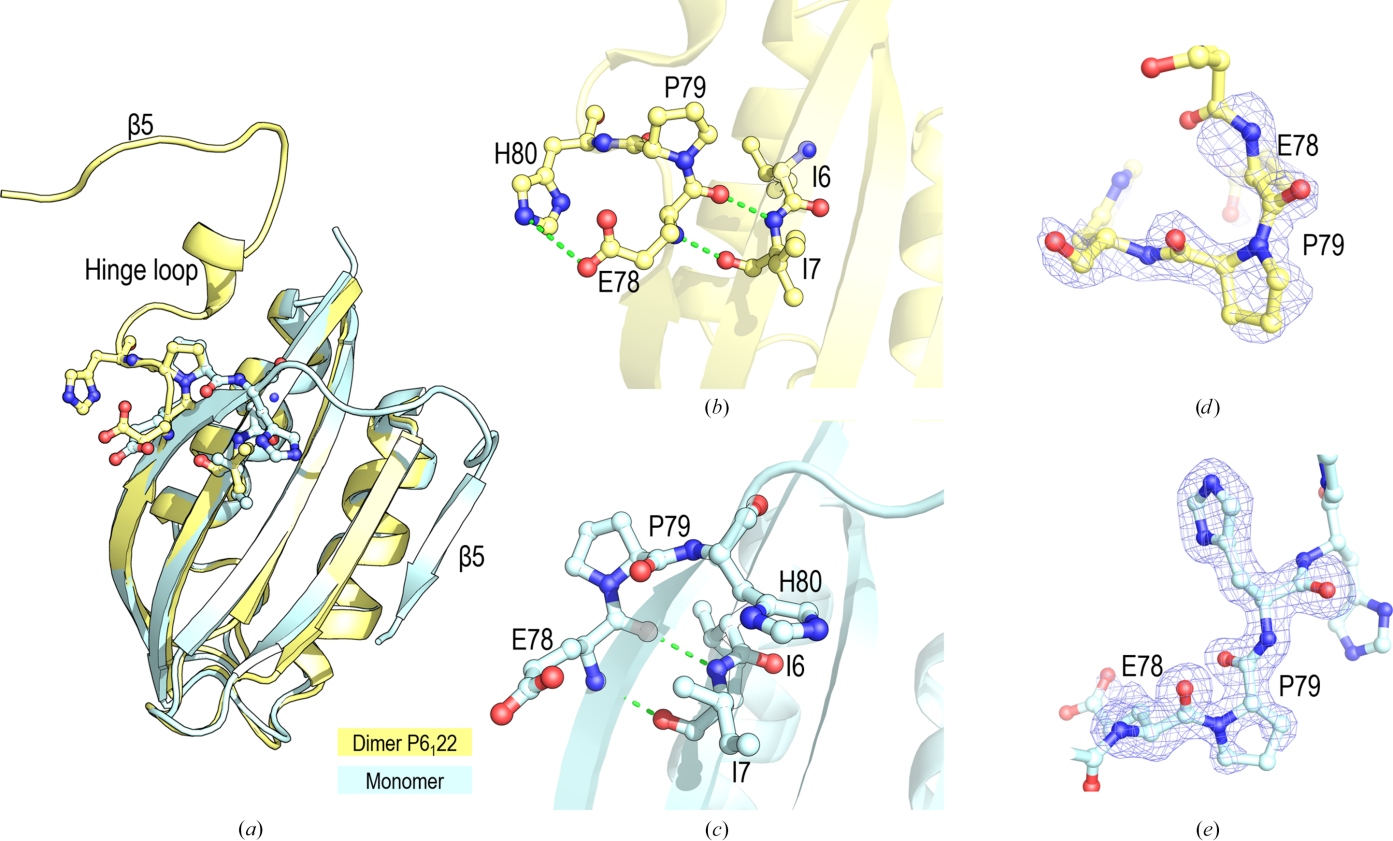
(*a*) Superposition of the protomers of the monomeric and dimeric forms of EcoAcP. Pro79 is in the (*b*) *cis* (PDB entry 9sv2) and (*c*) *trans* (PDB entry 9sv1) conformations. Two hydrogen bonds between the backbone atoms of Glu78-Pro79 and those of residues Ile6-Ile7 in β1 are shown as green dashed lines. The *trans* conformation in the dimer allows interaction between the Glu78 and His80 side chains. (*d*, *e*) *F*_o_ − *F*_c_ omit maps contoured at 3σ of the (*d*) *cis* and (*e*) *trans* Glu78-Pro79 peptide. Residues 78–80 were omitted to generate the omit map.

An interesting feature of both intertwined structures, the hexagonal (PDB entry 9sv2) and orthorhombic (PDB entry 8bv9) polymorphs, is the presence of the Glu78-Pro79 peptide unit in a *cis* conformation. Pro79 is the first residue of loop L5 and, in the monomeric structure, the Glu78-Pro79 peptide unit exhibits a *trans* conformation in both chains in the asymmetric unit (Fig. 5 ▸). At room temperature, *cis*-Pro exhibits slow isomerization to the *trans* state, with a rate of approximately 10^−3^–10^−2^ s^−1^, depending on the nature of the adjacent residues. Indeed, this isomerization can be the rate-limiting step in protein folding, as most non-native *cis*-Pro bonds in the unfolded protein must isomerize to the native *trans* conformation for folding to proceed (Alderson *et al.*, 2018 ▸; Wedemeyer *et al.*, 2002 ▸). The presence of Pro79 in the *cis* conformation would lead to the formation of stable on- or off-pathway intermediates, as previously described for the Cys5Ala/Cys49Ala variant of EcoAcP (Parrini *et al.*, 2008 ▸). The backbone N atom of His80 forms a hydrogen bond to the carbonyl O atom of Cys5 in the monomeric structure (Supplementary Table S1), but this bond is absent in the intertwined dimer structure. It is worth noting that the EcoAcP Pro79 residue is absent in other AcPs (Fig. 1 ▸). Besides, the characteristic higher fold rate of EcoAcP, compared with other AcPs, has been attributed to formation of the disulfide bond between Cys5 and Cys49.

### 3.4. Comparison of the monomeric and dimeric forms of EcoAcP

Superposition of backbone residues 1–78 of the monomeric polymorph (PDB entry 9sv1) and the hexagonal polymorph of the intertwined dimer (PDB entry 9sv2) structures of EcoAcP results in a backbone r.m.s.d. value of 0.69 Å. A comparison of the φ and the ψ values of the residues in the hinge loop, Pro79–Thr87, shows significant deviations between the monomeric and dimeric structures (Table 3 ▸), but also when compared with the intertwined structure of the orthorhombic polymorph (PDB entry 8bv9). Nevertheless, the most notable difference between the monomeric and dimeric structures is the formation of a 3_10_-helix (residues 81–86) in the hinge loop that leads to formation of the dimeric structure. Previously, the formation of new secondary-structure elements in the hinge-loop region has been observed in other proteins following 3D-DS (Salinas-Garcia *et al.*, 2025 ▸; Cámara-Artigas *et al.*, 2014 ▸; Xie *et al.*, 2021 ▸; Cámara-Artigas, 2016 ▸). Although most of the time the secondary structure formed by the hinge loop residues is a β-strand, a short 3_10_-helix has been observed in the hinge region of other 3D-domain-swapped structures. Previously, a comparison of the intertwined dimer structure and the monomeric structure obtained by NMR (PDB entry 2gv1; Pagano *et al.*, 2006 ▸) revealed differences in loop L4, which connects α2 to β4 (residues Trp61–Arg71). These differences are also observed among the ensemble of NMR models of EcoAcP and can be attributed to the flexibility of this loop in its unligated form (Supplementary Fig. S2).

**Figure 6:**
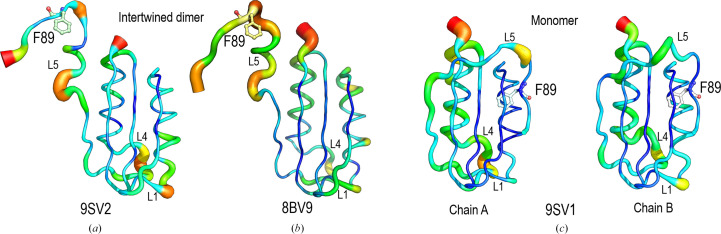
*B*-factor representations of the intertwined dimer of EcoAcP in (*a*) the hexagonal space group (PDB entry 9sv2), (*b*) the orthorhombic space group (PDB entry 8bv9) and (*c*) chains *A* and *B* of the monomeric structure (PDB entry 9sv2). Phe94 is shown in stick representation.

The *B*-factor values reflect the flexibility of the crystallo­graphic structure, and a *B*-factor putty representation of the monomeric and intertwined structures shows lower values in α1 and three β-strands (β1, β2 and β3) (Fig. 6 ▸). In both intertwined dimer structures the fifth β-strand exhibits higher *B* factors, especially in the orthorhombic polymorph. Also, the hinge loop L5 exhibits high *B* factors, indicating its flexibility. Indeed, this region exhibits different conformations in the two monomer chains in the asymmetric unit, and in the two intertwined dimer structures, as well as a diverse hydrogen-bond network (Supplementary Tables S1 and S2). In PhoAcP (PDB entry 1w2i), L5 is stabilized predominantly by an extensive network of salt bridges and hydrogen bonds (Cheung *et al.*, 2005 ▸). In the monomeric EcoAcP structure, the backbone atoms of Asp88 and Gly34 form a hydrogen bond, while in both intertwined structures this bond is established between residues of different chains. Besides, in the intertwined dimers, favoured by the *cis*-Pro79 conformation, the His80 and Glu78 side chains are placed at a distance compatible with a salt bridge (∼4 Å), whereas in the monomeric structure the distance between these residues is ∼7 Å (Figs. 5 ▸*b* and 5 ▸*c*). It is worth mentioning that crystals of the intertwined dimers were obtained at pH 4.8, while those of the monomeric form were obtained at pH 7.5, where His80 is unprotonated. The new 3_10_-helix (residues 81–86) in the hinge loop enables hydrogen-bond interactions that are absent in the monomeric form (Fig. 5 ▸*a*). The hydrogen-bond interactions of the residues that comprise L5 and adjacent residues (Glu78–Phe89) of the monomeric and dimeric structures are compiled in Supplementary Tables S1 and S2 and the salt bridges in Supplementary Table S3.

## 4. Discussion

### 4.1. Folding and formation of 3D-domain-swapped dimers in EcoAcP

AcPs have been the focus of many folding studies (Dagan *et al.*, 2013 ▸; Taddei *et al.*, 1994 ▸; Chiti *et al.*, 1998 ▸; Vendruscolo *et al.*, 2001 ▸; Parrini *et al.*, 2008 ▸; Chiti, Webster *et al.*, 1999 ▸; Chiti, Taddei *et al.*, 1999 ▸; van Nuland *et al.*, 1998 ▸; Bemporad *et al.*, 2004 ▸), some of them focused on the propensity of this small enzyme to form amyloid fibrils (Chiti, Webster *et al.*, 1999 ▸; Chiti & Dobson, 2017 ▸; Capanni *et al.*, 2004 ▸, Chiti *et al.*, 2000 ▸, 2001 ▸). Many proteins that form amyloids have also been described as 3D-DS oligomers (Cámara-Artigas, 2016 ▸; Janowski *et al.*, 2001 ▸; Staniforth *et al.*, 2001 ▸; Kumar *et al.*, 2023 ▸). Although the molecular basis of the relationship between 3D-DS and amyloid formation is not fully understood, both processes involve opening the protomer to form the characteristic oligomers. The availability of a 3D-DS structure and comparison with the monomeric structure would facilitate understanding of the molecular basis of oligomer formation. Although EcoAcP is the first AcP where the formation of 3D-DS oligomers has been described, some small oligomers have been described to precede amyloid formation in the AcP from human muscle (Chiti *et al.*, 2000 ▸). Also, the AcP from *S. solfataricus* (SsoAcP) has been shown to fold through the accumulation of a partially folded species (Bemporad *et al.*, 2004 ▸). Moreover, the N-terminal domain of HypF from *E. coli* (HypF-N; PDB entry 1gxt), a 91-residue protein module sharing the same folding topology and significant sequence identity with EcoAcP, was found to collapse into a partially folded intermediate before reaching the fully folded conformation (Calloni *et al.*, 2003 ▸).

AcPs, like most small single-domain proteins, fold in a two-state manner, with only the fully unfolded and native states being highly populated during folding. It has been proposed that a primary determinant of the folding rate for a small protein is the relative contact order (RCO), which is the average distance in sequence between residues that form an interaction in the native state, normalized to the total number of residues (Plaxco *et al.*, 1998 ▸). Indeed, the slow folding of AcPs has also been attributed to the abundance of long-range interactions, which results in a high RCO value. The RCO of this small protein contributes to the high folding barrier between the folded and unfolded states and, in some cases, has been proposed to form a partially formed hydrophobic nucleus (Parrini *et al.*, 2008 ▸). However, among AcPs, EcoAcP is an exception as its folding is accelerated by the presence of a disulfide bond between Cys5 and Cys49 (Parrini *et al.*, 2008 ▸). This way, EcoAcP folds over three orders of magnitude faster than expected based on its RCO and hydrophobicity, whereas the mutant, in which the two cysteine residues have been replaced by alanines, folds much slower, in accordance with its RCO and hydrophobicity. The monomeric structure reported in this work also points to a relevant role for the salt bridge between Arg20 and the carboxyl-terminal group of Arg92, which might also contribute to the RCO value. This salt bridge is absent in the dimeric structure of the hexagonal polymorph, where the cloning-artefact residue Leu93 has been modelled (Leu93 and Glu94 in the previous dimeric structure, the orthorhombic polymorph PDB entry 8bv9).

**Figure 7:**
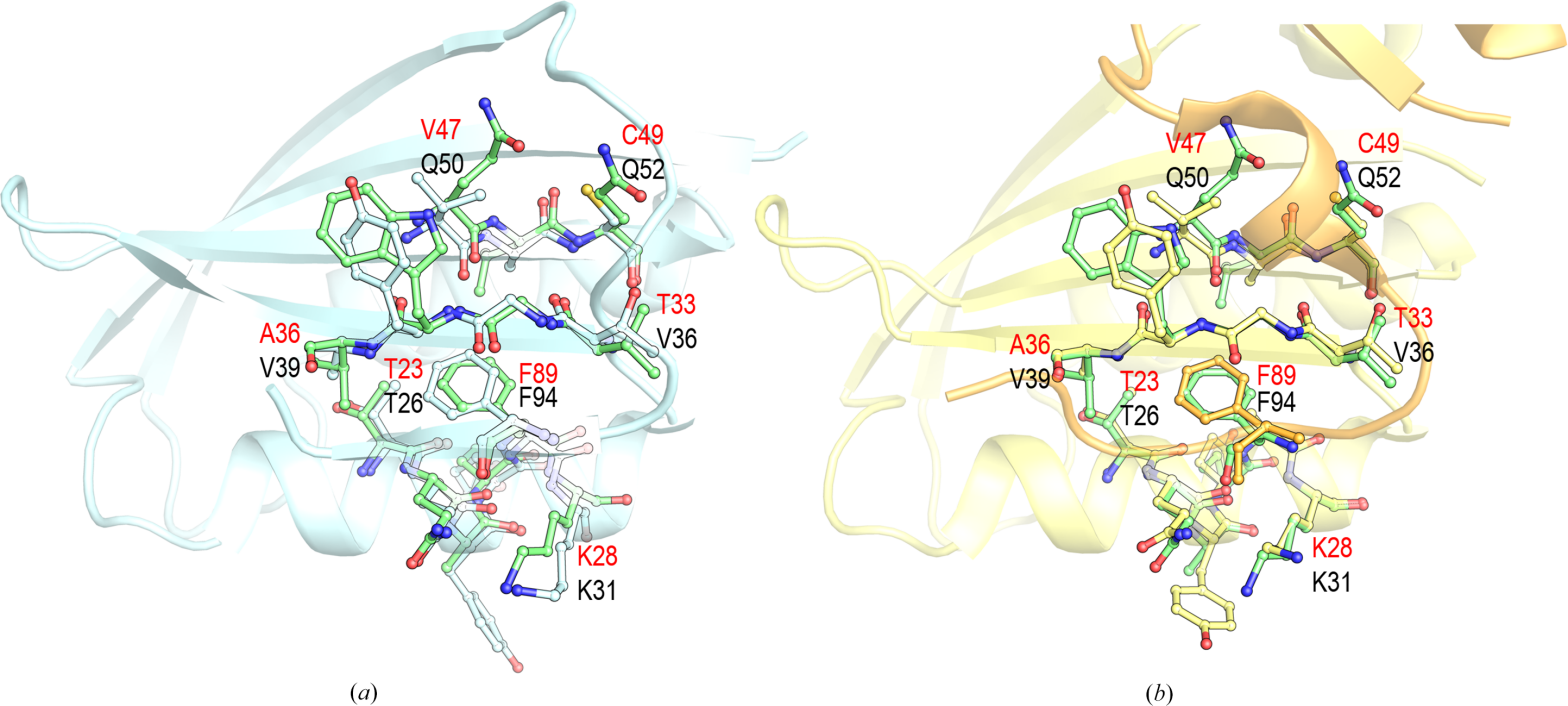
Superposition of HsaAcP (PDB entry 2vh7) with (*a*) the monomeric (PDB entry 9sv1) and (*b*) the dimeric (PDB entry 9sv2) structures of EcoAcP. In HsaAcP, Phe94 forms a critical contact network in the folding transition state with residues 26–31 in α1, 36–39 in β2 and 50–52 in β3. The equivalent contact network of Phe89 in EcoAcP is accomplished by residues 23–28 in α1, 33–36 in β2 and 47–49 in β3. In the dimeric structure, contacts are established between residues 23–28 in α1, 33–36 in β2, 47–49 in β3 and Phe89 in a symmetry-related molecule that generates the intertwined dimer. Residues belonging to HsaAcP and EcoAcP are labelled in black and red, respectively.

Folding studies with small model proteins suggest that transition states are conserved between proteins with the same native fold (Chiti, Taddei *et al.*, 1999 ▸; Martinez & Serrano, 1999 ▸). A mutational analysis of *Homo sapiens* muscle AcP (HsamAcP, UniProt code P14621) reveals that the residues with higher φ values are Tyr11, Pro54 and Phe94, indicating that these residues form native contacts in the transition state (Vendruscolo *et al.*, 2001 ▸). In contrast, residues Ile86 and Leu89, belonging to L5, show near-zero φ-values. Phe94 is conserved among the AcP sequences (Fig. 1 ▸). However, Tyr11 and Pro54 are replaced by Ala8 and Glu51 in EcoAcP, respectively. The conserved Phe94 (Phe89 in EcoAcP) in all AcP sequences points to the significance of this residue in the folding of this small protein. This phenylalanine is located in β5 and is fully buried in the hydrophobic core of the monomeric EcoAcP (ASA of 2.8 and 6.1 Å^2^ for chains *A* and *B*, respectively) and the dimeric structure (ASA of 3.6 Å^2^). The comparison of the crystal structures of the monomeric and dimeric EcoAcP with the crystal structure of HsaAcP (PDB entry 2vh7; Lam *et al.*, 2011 ▸) is shown in Fig. 7 ▸. Phe94 forms a critical contact network with residues 26–31 in α1, 36–39 in β2 and 50–52 in β3, which are likely key to the folding transition state. These contacts are conserved in the native state of EcoAcP as residues 50–52 in α1, 33–36 in β2 and 46–48 in β3 in both the monomer and the intertwined dimer (Fig. 7 ▸). However, the *B*-factor putty representation (Fig. 6 ▸) clearly shows the highest *B* factors in β5 and L5 for both intertwined dimer structures.

Interestingly, comparison of the monomeric and dimeric structures of EcoAcP and HsaAcP (PDB entry 2vh7) shows residue Gln52 in β3 located at the same position as Cys49. This cysteine forms a disulfide bond with Cys5, which has been demonstrated to be critical for the folding rate of EcoAcP, as it significantly impacts the RCO (Parrini *et al.*, 2008 ▸). It is generally accepted that disulfide bonds play a crucial role in maintaining the structural integrity and stability of proteins. Comparison of the folding rate of EcoAcP with those of other AcPs demonstrated that its folding rate is higher than expected. The presence of a disulfide bond in the core of the protein may restrict the folding pathway by bringing residues of the folding nucleus into proximity, thereby facilitating folding to the native state. Moreover, a study using the Cys5Ala/Cys49Ala double mutant shows a deviation from the two-state model at low GdnHCl concentrations, attributed to the accumulation of a partially folded state during folding. Because of the salt condition of GdnHCl, a role of ionic strength in the folding of EcoAcP cannot be ruled out. Indeed, EcoAcP requires high concentrations of GdnHCl to achieve complete denaturation, and non-ionic chaotropic agents, such as urea, are ineffective at any concentration (Ramazzotti *et al.*, 2006 ▸). Besides the effect of the disulfide bond on the folding rate of EcoAcP, the presence of the salt bridge between Arg20 and the carboxyl-terminal moiety of Arg92 would also be important for the folding of this AcP. This salt bridge provides EcoAcP with characteristics of thermophilic AcPs (Lam *et al.*, 2011 ▸). Moreover, this salt bridge appears to be crucial for opening the hinge loop, and its absence favours the formation of the intertwined dimer. Otherwise, electrostatic interactions at the open interface of the intertwined dimer would also explain its stabilization. Indeed, we cannot rule out that stabilizing electrostatic interactions contribute to the dimerization of EcoAcP.

### 4.2. Proline isomerization

The proline ring structure restricts the conformation of proline itself and also the conformation of the preceding residue (MacArthur & Thornton, 1991 ▸). Most protein structures exhibit either the *cis* or the *trans* isomer due to conformational restraints resulting from close packing of side chains in the hydrophobic core, although there are exceptions to this general finding (for example, staphylococcal nuclease; Evans *et al.*, 1987 ▸). Proline *cis*–*trans* isomerization is a slow process that can affect the protein folding rate (Alderson *et al.*, 2018 ▸). Indeed, in human muscle AcP the slow folding rate of a minor fraction of the unfolded protein has been attributed to a *cis*–*trans* prolyl-isomerization phase (van Nuland *et al.*, 1998 ▸). In 3D-DS, proline *cis*–*trans* isomerization can act as a conformational gatekeeper, favouring protomer opening and facilitating intermolecular interactions between partially unfolded protomers to form domain-swapped oligomers (Miller *et al.*, 2010 ▸). A systematic analysis of proteins with 3D-DS structures found that over 40% of these proteins contain at least one proline residue in the hinge loop (Huang *et al.*, 2018 ▸). The role of these proline residues has been examined in several proteins (Huang *et al.*, 2018 ▸; Bergdoll *et al.*, 1997 ▸; Di Donato *et al.*, 1994 ▸; Han *et al.*, 2002 ▸; Schymkowitz *et al.*, 2000 ▸; Barrientos *et al.*, 2002 ▸; Rousseau *et al.*, 1998 ▸; Weininger *et al.*, 2009 ▸). The presence of *cis*-proline in the intertwined dimer of EcoAcP, but not in the monomeric form, suggests a role for *cis*–*trans* isomerization of Pro79 in the formation of the domain-swapped dimer. In EcoAcP, Pro79 is located at the beginning of the long hinge loop (Pro79–Asp88), and the literature provides evidence for the role of *cis*-proline in facilitating the formation of domain-swapped oligomers. This way, a non-native *cis* peptide bond in Pro90 in the hinge loop of p13suc has been proposed to be critical for the formation of the intertwined dimer of this protein, and the mutant in which this proline has been mutated to alanine does not dimerize (Rousseau *et al.*, 1998 ▸). Another example is stefin B, where the peptide bond of Pro74, conserved throughout the cystatin family, is in the *cis* conformation in the domain-swapped oligomer, in contrast to the *trans* conformation in the monomeric form (Jenko Kokalj *et al.*, 2007 ▸). However, many domain-swapped proteins lack proline residues in their hinge loops (Huang *et al.*, 2018 ▸). Thus, although proline *cis*–*trans* isomerization has been demonstrated to play a critical role in the formation of intertwined oligomers, other factors must also be considered.

## 5. Conclusions

In this work, the structures of monomeric and dimeric EcoAcP highlight the advantage of using this small enzyme not only as a good model for studying protein folding, but also for exploring the molecular basis of the complex and, to date, still poorly understood process of 3D domain-swapping. High-resolution structures reveal interactions at the open interface, favouring the formation of the intertwined dimer over the monomeric form. As the closed interface is conserved, new interactions in the open interface and lower conformational energy might favour the intertwined oligomer (Ding *et al.*, 2002 ▸). Besides, transient interactions can also displace the folding equilibrium to monomer or dimer formation (Assar *et al.*, 2016 ▸). Analysis of new interactions at the open interface reveals the formation of a short 3_10_-helix in residues of the hinge loop, enabling new interactions absent in the monomeric form. It is worth noting that EcoAcP exhibits characteristics that distinguish its folding process from that of other AcPs. Indeed, EcoAcP showed a faster folding rate than that observed for other AcPs, attributed to the disulfide bond between Cys5 and Cys49. The presence of this disulfide bond significantly reduces the conformational entropy and RCO of EcoAcP. In the intertwined dimer, the hydrogen bond between the backbone atoms of Cys5 and His80, next to Pro79, is lost. This proline residue is present in a *cis* conformation in both intertwined structures obtained from different polymorphs, while it shows a *trans* conformation in the monomeric form. Interestingly, Pro79 is the first residue of L5, the hinge loop providing the interchange of secondary structure. In the protomer of EcoAcP, the synergy between loss of the hydrogen bond between the backbone atoms of Cys5 and His80 and the *cis* conformation of Pro79 would favour the opening of L5 and formation of the intertwined dimer. Moreover, Cys49 is part of the network of interactions surrounding Phe89, one of the key residues forming native contacts in the transition state (Fig. 7 ▸). Finally, an in-depth analysis of domain-swapped structures could be a powerful tool for studying long-range interactions that participate in the protein folding process and secondary-structure elements already present in the early folding stages (Salinas-Garcia *et al.*, 2025 ▸).

## 6. Related literature

The following reference is cited in the supporting information for this article: Cock *et al.* (2009 ▸).

## Supplementary Material

PDB reference: acylphosphatase from *Escherichia coli*, monomer, 9sv1

PDB reference: intertwined dimer, 9sv2

Supplementary Tables and Figures. DOI: 10.1107/S2059798326001774/nz5020sup1.pdf

## Figures and Tables

**Table 1:** Data collection and processing Values in parentheses are for the outer shell.

	EcoAcP monomer	EcoAcP intertwined dimer
PDB entry	9sv1	9sv2
Diffraction source	XALOC, ALBA	ID30A-3, ESRF
Wavelength (Å)	0.98	0.98
Temperature (K)	100	100
Space group	*P*2_1_2_1_2_1_	*P*6_1_22
*a*, *b*, *c* (Å)	28.30, 48.58, 103.04	90.79, 90.79, 40.28
α, β, γ (°)	90, 90, 90	90, 90, 120
Resolution range† (Å)	19.92–1.55 (1.58–1.55)	19.77–1.95 (2.00–1.95)
Total No. of reflections	76715 (1376)	24365 (1741)
No. of unique reflections	20465 (757)	6191 (431)
Completeness (%)	95.8 (72.9)	83.2 (85.2)
Multiplicity	3.7 (1.8)	5.8 (1.2)
〈*I*/σ(*I*)〉‡	12.8 (1.3)	5.8 (1.2)
*R*_merge_	0.05 (0.54)	0.13 (0.84)
CC_1/2_	0.99 (0.59)	0.99 (0.60)
Overall *B* factor from Wilson plot (Å^2^)	17.0	28.8

† The resolution cutoff was determined based on the CC_1/2_ criterion (Karplus & Diederichs, 2012 ▸).

‡ Average *I*/σ(*I*), as defined in *AIMLESS*.

**Table 2:** Structure solution and refinement Values in parentheses are for the outer shell.

	EcoAcP monomer	EcoAcP intertwined dimer
PDB entry	9sv1	9sv2
Resolution range (Å)	19.92–1.55 (1.63–1.55)	19.77–1.95 (2.46–1.95)
No. of reflections, working set	19381 (2203)	5899 (2968)
No. of reflections, test set	1036 (107)	290 (140)
Final *R*_cryst_	0.16 (0.29)	0.21 (0.23)
Final *R*_free_	0.20 (0.31)	0.26 (0.30)
No. of non-H atoms		
Protein	1447	717
Ligand	16	58
Water	181	34
Total	1644	787
R.m.s. deviations		
Bond lengths (Å)	0.011	0.014
Angles (°)	1.14	1.25
Average *B* factors (Å^2^)		
Overall	21.7	30.2
Protein	20.9	29.4
Ligand	21.1	43.7
Water	28.6	34.8
Ramachandran plot		
Most favoured (%)	98.3	96.6
Allowed (%)	1.7	3.4
Rotamer outliers (%)	0	0
Clashscore	1.4	0.7

**Table 3:** φ and ψ values of the residues belonging to the hinge loop

	Intertwined dimer				Monomer	
PDB code	8bv9		9sv2		9sv1	
					Chain *A*	Chain *B*
Residue	φ (°)	ψ (°)	φ (°)	ψ (°)	φ (°)	ψ (°)
Glu78	−90.9	−43.9	−135.3	145.9	−144.2/−142.3	156.5/155.1
Pro79	−80.8	96.5	−76.2	161.9	−62.7/−61.6	153.1/151.7
His80	−64.4	−24.1	−65.3	−30.7	−140.2/−134.5	142.4/143.2
His81	−125.5	94.3	−125.4	82.1	−130.7/−127.4	78.9/91.6
Pro82	−64.5	−13.5	−63.1	−23.3	−85.8/−69.2	173.9/155.9
Ser83	−71.0	−13.9	−62.9	−22.1	−68.1/−78.1	170.8/76.5
Gly84	−68.9	−19.1	−66.6	−9.5	−106.8/97.8	17.3/148.0
Glu85	−75.1	−14.3	−65.2	−15.5	−70.8/−59.2	−24.2/145.1
Leu86	−112.1	−10.3	−110.0	168.1	−83.9/−96.8	114.7/143.6
Thr87	−75.5	136.9	81.2	−55.1	−102.2/−115.9	0.1/−8.4
Asp88	−51.1	147.0	−149.9	−173.7	−128.9/−145.4	−174.8/−179.9
