# Supplementary material for: Molecular analysis of 3D domain swapping in the acylphosphatase from Escherichia coli
Source: Acta Crystallogr D Struct Biol. 2026 Mar 19;82(Pt 4):336–47. doi: 10.1107/S2059798326001774 (PMC13044899; doi:10.1107/S2059798326001774)
Supplement: Supplementary file 1 [file d-82-00336-sup1.pdf]

STRUCTURAL BIOLOGY

Volume 82 (2026)

Supporting information for article:

Molecular analysis of 3D domain swapping in the acylphosphatase from *Escherichia coli*

Sergio Martínez-Rodríguez, Jose A. Gavira, M. Carmen Salinas-Garcia, Montserrat Andujar-Sánchez and Ana Camara-Artigas

Table S1 Main chain hydrogen bond network in the monomeric and dimeric structures. Sequence comprising L5 and adyacent residues (Glu78-Phe89)

Hbond accept (CO)	Hbond donor (NH)	Distance (Å)	Hbond angle	Hbond energy (kcal/mol)
**9SV1**				
CYS 5A	HIS 80A	2.4	107.9	-1.39
ILE 7A	GLU 78A	2.1	173.7	-2.03
GLU 78A	ILE 7A	2.1	174.5	-2.12
ASP 88A	GLY 34A	2.2	128.1	-1.8
CYS 5B	HIS 80A	2.3	110.8	-1.50
ILE 7B	GLU 78B	2.0	174.8	-2.03
GLU 78B	ILE 7B	2.0	176.5	-2.3
ASP 88B	GLY 34B	2.0	125.1	-2.1
**9SV2**[1]				
ILE 7A	GLU 78A	1.8	176.2	-2.6
GLU 78A	ILE 7A	2.0	170.8	-2.4
HIS 81A	GLY 84A	2.04	143.28	-1.8
PRO 82A	GLU 85A	2.01	129.54	-1.8
SER 83A	LEU 86A	2.54	118.62	-1.1
**ASP 88A** [2]	**GLY 34B**	**1.99**	**128.54**	**-2.3**

[1] In the intertwined dimer, chain B has been generated by symmetry operators.

[2] Interactions between different chains in the intertwined dimer are marked in bold.

Table S2 Sidechain hydrogen bond network in the monomeric and dimeric structures. Sequence comprising L5 and adyacent residues (Glu78-Phe89)

Sidechain donor		Sidechain acceptor		Distance (Å)
**9SV1**				
GLN 24A	NE2--CD	PHE 89A	O--C	2.9
SER 83A	OG--CB	GLU 85A	OE1--CD	2.4
ARG 90A	NE--CZ	ASP 88A	OD1--CG	2.9
ARG 90A	NE--CZ	ASP 88A	OD2--CG	3.3
GLN 24A	NE2--CD	PHE 89A	O--C	2.9
THR 33B	OG1--CB	LEU 86B	O--C	3.2
HIS 81B	N --H	HIS 80B	ND1--CG	3.5
HIS 81B	ND1--CG	HIS 80B	O--C	3.4
ASP 88B	N --H	THR 87B	OG1--CB	3.5
ARG 90B	NE--CZ	ASP 88B	OD1--CG	2.9
GLN 24B	NE2--CD	PHE 89B	O--C	2.9
**9SV2**[1]				
**GLN 24A**[2]	**NE2--CD**	**PHE 89B**	**O--C**	**3.0**
HIS 80A	ND1--CG	SER 77A	O--C	3.4
HIS 81A	ND1--CG	HIS 80A	O--C	3.3
GLU 85A	OE2--CD	CYS 5A	O--C	2.5
PHE 89A	N --H	ASP 88A	OD1--CG	3.0
ARG 90A	NH2--CZ	GLY 84A	O--C	2.9
ARG 90A	NH2--CZ	LEU 86B	O--C	3.0

[1] In the intertwined dimer, chain B has been generated by symmetry operators.

[2] Interactions between different chains in the intertwined dimer are marked in bold.

Table S3 Salt bridges observed in the monomeric and dimeric structures of EcoAcP

9SV1		
Chain A	Chain A	Distance (Å)
GLU26(OE1)	ARG29(NH2)	2.8
ASP88(OD1)	ARG90(NH2)	3.9
ARG20(NH1)	ARG92(OXT)	2.8
Chain B	Chain B	
GLU26(OE2)	ARG29(NH2)	2.5
GLU73(OE2)	ARG71(NH2)	3.8
ASP88(OD1)	ARG90(NH2)	3.7
ARG20(NH1)	ARG92(OXT)	2.8
9SV2		
Chain A	Chain A	Distance (Å)
GLU26(OE1)	ARG29(NH1)	3.2
GLU51(OE1)	LYS3(NZ)	3.1
GLU73(OE2)	ARG71(NH1)	2.8
GLU73(OE2)	ARG71(NH2)	3.9
GLU78(OE2)	HIS80(NE2)	3.7
ASP88(OD1)	ARG90(NH2)	3.1

Salt bridges in the monomeric and dimeric structures were identified using a BioPython-based script that calculated distances between charged residues and selected interactions with interatomic distances below 4 Å (Cock *et al.*, 2009).

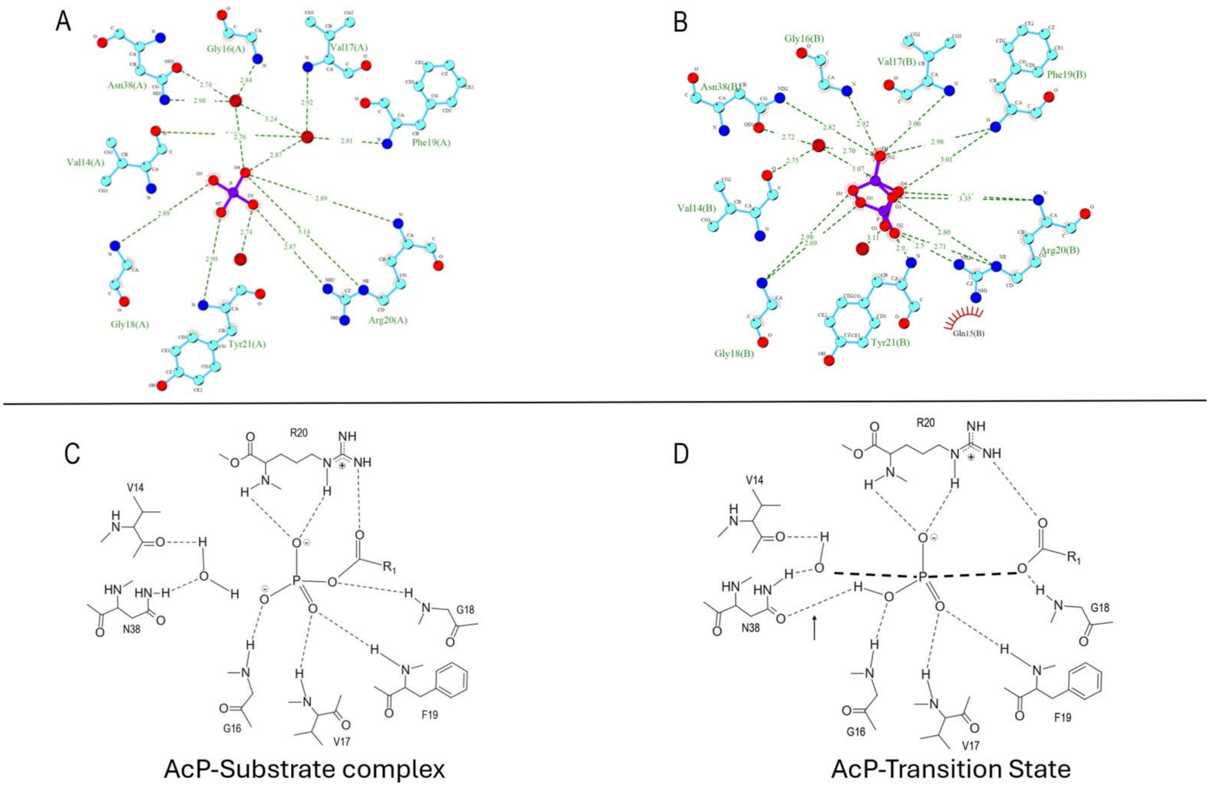

Figure S1 Representation of the active site of the two chains of the monomeric structure. (A) Chain A shows the presence of a single phosphate bound, meanwhile, (B) in chain B, a phosphate ion has been modelled in two alternate conformations. Catalytic mechanism proposed for the acylphosphatases: (C) substrate-enzyme complex, and (D) transition state (reproduced from Cheung YY et al. *Crystal structure of a hyperthermophilic archaeal acylphosphatase from Pyrococcus horikoshii: structural insights into enzymatic catalysis, thermostability, and dimerization*. Biochemistry. 2005 Mar 29;44(12):4601-11. doi: 10.1021/bi047832k)

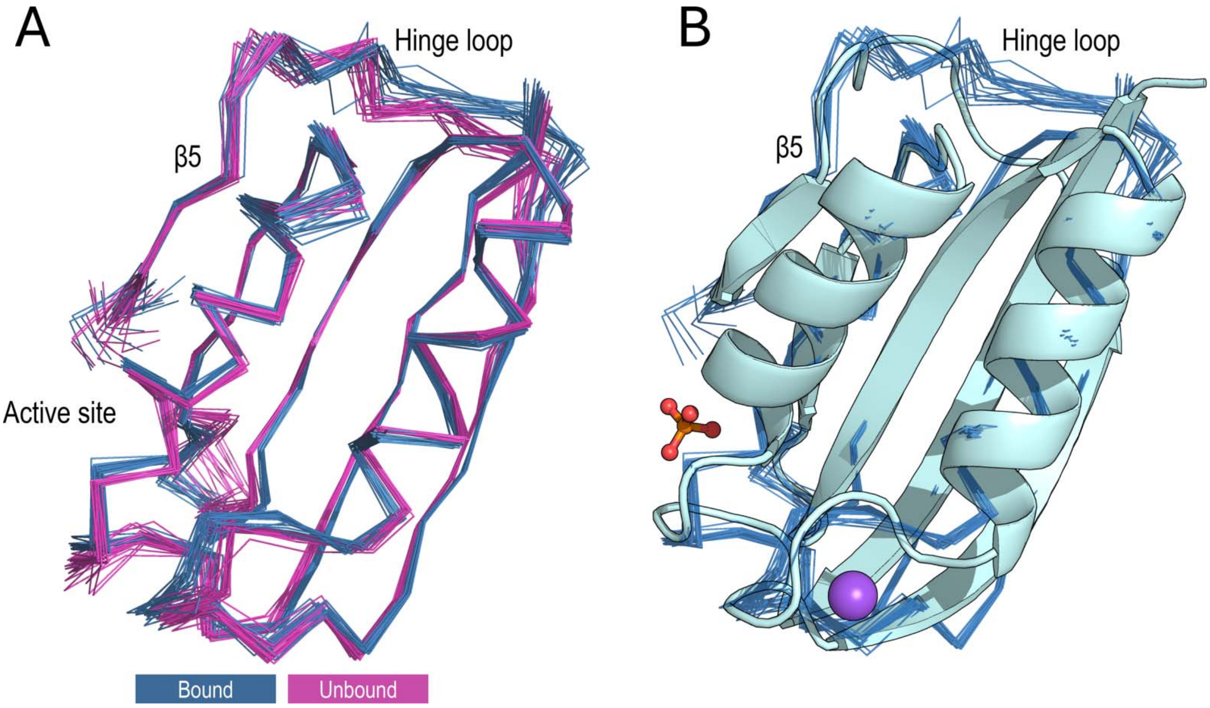

Figure S2 (A) Superposition of the BsuAcP NMR structures of the phosphate-bound (blue) (PDB entry 2HLT) and free (magenta) enzyme (PDB entry 2HLU). (B) Superposition of the BsuAcP NMR structures of the phosphate-bound (blue) and the crystallographic structure of the monomeric EcoAcP (cyan, chain A).
